# Resilience as a Mediator in a Web-Based Intervention (MINDxYOU) to Reduce Stress Among Health Care Professionals: Stepped-Wedge Cluster Randomized Trial

**DOI:** 10.2196/82905

**Published:** 2026-02-09

**Authors:** Gloria Guerrero-Pertiñez, Vera Carbonell-Aranda, Gloria Pérez-Guerrero, Jonathan Joseph Dawood-Hristova, Adrián Pérez-Aranda, Selene Fernández-Martínez, Alicia Monreal-Bartolomé, Alberto Barceló-Soler, Javier García-Campayo, Yolanda López del Hoyo, Jesús Herrera-Imbroda, Jessica Marian Goodman-Casanova, Jose Guzman-Parra

**Affiliations:** 1 Unidad de Gestión Clínica de Salud Mental, Hospital Regional Universitario de Málaga, Instituto de Investigación Biomédica de Málaga (IBIMA)-Plataforma Bionand, Plaza del Hospital Civil, s/n, Málaga, 29009, Spain, 34951290309; 2 Institut d'Investigació Sanitària Pere Virgili, Tarragona, Spain; 3 Department of Clinical and Health Psychology, Universitat Autònoma de Barcelona, Barcelona, Spain; 4 CIBER of Epidemiology and Public Health (CIBERESP), Madrid, Spain; 5 Faculty of Medicine, University of Zaragoza, Zaragoza, Spain; 6 Department of Psychology and Sociology, University of Zaragoza, Zaragoza, Spain; 7 Institute of Health Research of Aragon, Zaragoza, Spain; 8 Research Network on Chronicity, Primary Care and Health Promotion (RICAPPS), Zaragoza, Spain

**Keywords:** mindfulness, resilience, eHealth, health professionals, stress, mental health

## Abstract

**Background:**

The mechanisms through which mindfulness and third wave–based digital programs exert their effects on stress reduction remain poorly understood. Identifying these mediators is essential to optimize their implementation, particularly in health care settings. This approach is particularly relevant for health care professionals, who are constantly exposed to high levels of emotional demands, work overload, and risk of burnout, especially in the aftermath of the COVID-19 pandemic. Despite the growing need for scalable and accessible mental health support in this population, such digital programs remain scarce and underused.

**Objective:**

The primary aim of this study was to analyze the psychological mechanisms through which the MINDxYOU online program may contribute to stress reduction among health care professionals, focusing on a mediation model. Specifically, we explored if variables such as resilience and facets of mindfulness, compassion, and acceptance mediated the effects of the intervention on perceived stress.

**Methods:**

In a stepped-wedge cluster randomized design, 357 health professionals from health centers in Aragon and Málaga, Spain, were recruited. They were divided into 6 clusters, 3 per region, and randomly assigned to one of the 3 sequences, each starting with a control phase and then transitioning to the intervention phase (the MINDxYOU program) after 8, 16, or 24 weeks. This self-guided, web-based program, designed to be completed over 8 weeks, included weekly contact (via WhatsApp, call, or email) from the research team to promote adherence. Participants were assessed on the web every 8 weeks for 5 assessments. Perceived stress was the study’s primary outcome, with additional measures of clinical factors (anxiety, depression, and somatization) and process variables (resilience, mindfulness, compassion, and acceptance). Mediation models using mixed-effects regressions and bootstrap resampling (1000 iterations) were applied to analyze the direct and indirect effects of the treatment on psychological outcomes.

**Results:**

Resilience emerged as the most consistent and significant mediator, exerting a relevant indirect effect on reducing stress (β=−1.41; *P*=.02), anxiety (β=−0.88; *P*=.03), and depression (β=−0.97; *P*=.01), even in multivariate models. Mindfulness facets such as observing, describing, and nonreacting also showed significant, albeit less consistent, mediating effects. In contrast, compassion and acceptance were weakly associated and did not play a significant mediating role.

**Conclusions:**

These results demonstrate resilience as the key psychological mediator. Strengthening resilience through online interventions appears to be a crucial pathway for reducing stress and emotional symptoms in this population. Specific mindfulness skills may also contribute to the intervention’s therapeutic effect, although with less robust evidence.

## Introduction

Health care professionals (HCPs) are consistently exposed to high levels of emotional demands, work overload, and ethical stress, which significantly increase their risk of burnout and psychological distress, especially in the aftermath of the COVID-19 pandemic [1]. Emotional exhaustion and persistent stress among health care workers not only deteriorate their own mental health [2] but also negatively affect patient care, leading to reduced empathy, lower job performance, increased medical errors, and decreased patient satisfaction [3,4]. Promoting psychological well-being in this group is therefore not only an occupational health priority but also a critical factor for ensuring the quality, safety, and sustainability of health care delivery systems [5].

In this context, third wave psychotherapies, including acceptance and commitment therapy (ACT), mindfulness-based interventions, and compassion-based programs, have proven effective in enhancing emotional well-being and reducing symptoms of stress, anxiety, and depression [6-8]. These interventions target transdiagnostic psychological processes such as emotional regulation, acceptance of internal experience, and the cultivation of compassionate attitudes toward oneself and others [9,10], thereby fostering more flexible and adaptive coping strategies in the face of chronic occupational stress [11]. Third wave psychotherapies were originally developed for traditional face-to-face delivery, often in group settings. However, the rise of digitalization has significantly reshaped access to psychological interventions, enabling the development and implementation of eHealth programs grounded in third wave principles. These types of interventions have gained relevance as an accessible and effective alternative for promoting mental health [12]. One of their main advantages lies in their ability to overcome geographic and economic barriers, thus expanding access to these psychotherapies for broad and diverse populations [13].

Despite the growing evidence supporting the effectiveness of interventions based on third wave psychotherapy principles, the specific mechanisms underlying their therapeutic benefits still require further clarification [14,15]. Brief interventions based on third wave psychotherapy principles have demonstrated significant improvements in resilience, anxiety, and depression among nursing staff, and recent meta-analytic evidence confirms the relevance of these mechanisms in digital mental health programs [6]. Mindfulness practice, in particular, has been shown to enhance core psychological capacities such as present-moment awareness, resilience, acceptance, and compassion [10,16], all of which contribute to reducing emotional reactivity, rumination, and psychological distress. Within this framework, acceptance, a central process in ACT, has been identified as especially relevant in decreasing experiential avoidance, a transdiagnostic factor involved in many emotional disorders [17]. Supporting this, recent studies have found that ACT delivered in digital formats can significantly improve emotional regulation, cognitive flexibility, and stress coping in HCPs [17].

Some psychological skills appear to exert a more consistent impact than others, depending on the type of intervention, target population, or delivery format (in-person, hybrid, or online) [16], which affects their effectiveness as mechanisms of change. In particular, specific components of mindfulness such as observing, describing, and nonreacting have demonstrated significant mediating roles in improving psychological well-being [17]. In contrast, other skills such as acceptance or self-compassion have shown more variable results in terms of their explanatory power [18,19]. On its part, resilience, which is a key psychological resource particularly in emotionally demanding environments such as health care, has also been identified as a potential mediator of third wave psychotherapies’ effects on stress relief and health promotion [20-22]. While mindfulness, self-compassion, and acceptance have been proposed as mediators, findings are inconsistent across studies and contexts. In some cases, these variables significantly explain symptom reduction, whereas in others, their effects are weak or nonsignificant [6].

These theoretical and empirical discrepancies highlight the need for continued investigation into which psychological processes function as mechanisms of change, how they interact with one another, and under what contextual and individual conditions their effects are enhanced or constrained particularly within the realm of contemporary digital interventions. This study aims to analyze the mediating role of psychological variables in the effect of an online intervention based on third wave psychotherapy principles (MINDxYOU) [23,24] on HCPs. Specifically, it examines whether resilience, mindfulness, compassion, and acceptance mediate the effect of the intervention on the primary outcome, perceived stress, and on several secondary outcomes: depression, anxiety, and general psychological symptoms.

## Methods

### Participants and Procedure

Participants in this study were enrolled in the MINDxYOU project, a stepped-wedge cluster-randomized clinical trial. The study sample consisted of HCPs working in clinical settings in Málaga and Zaragoza (Spain). Inclusion criteria were (1) employment as a HCP (eg, physician, nurse, psychologist, and nursing assistant) or being in training in any health-related field; (2) aged between 18 and 70 years; (3) ability to understand Spanish; (4) digital literacy and access to a smartphone, tablet, or computer with internet connection; and (5) expected continued employment at the same workplace for the following 6 months. Candidates who met these criteria were also asked to provide their email address or phone number in the form so that the researchers could contact them later.

Between October 2022 and February 2023, those interested in participating underwent a telephone screening conducted by a mental health professional to assess the following exclusion criteria: (1) presence of a severe disorder affecting the central nervous system; (2) diagnosis of a severe mental illness according to the Mini-International Neuropsychiatric Interview (MINI) [25]; (3) presence of an uncontrolled medical condition or an infectious or degenerative disease; and (4) prior experience with third wave psychotherapies (eg, having participated in mindfulness courses during the previous year or having regularly engaged in formal meditation practice). Candidates who met the inclusion profile were sent an email with detailed information about the study and an attached informed consent form.

As detailed in the study protocol [23], the sample size was estimated at 180 participants, with 30 (16.7%) per cluster, based on an expected moderate effect of the intervention on the primary outcome, perceived stress [26]. However, the ambitious dissemination of the project attracted considerable interest from HCPs. A total of 357 individuals provided data, and 347 ultimately completed the baseline assessment.

### Intervention

**Figure 1.**
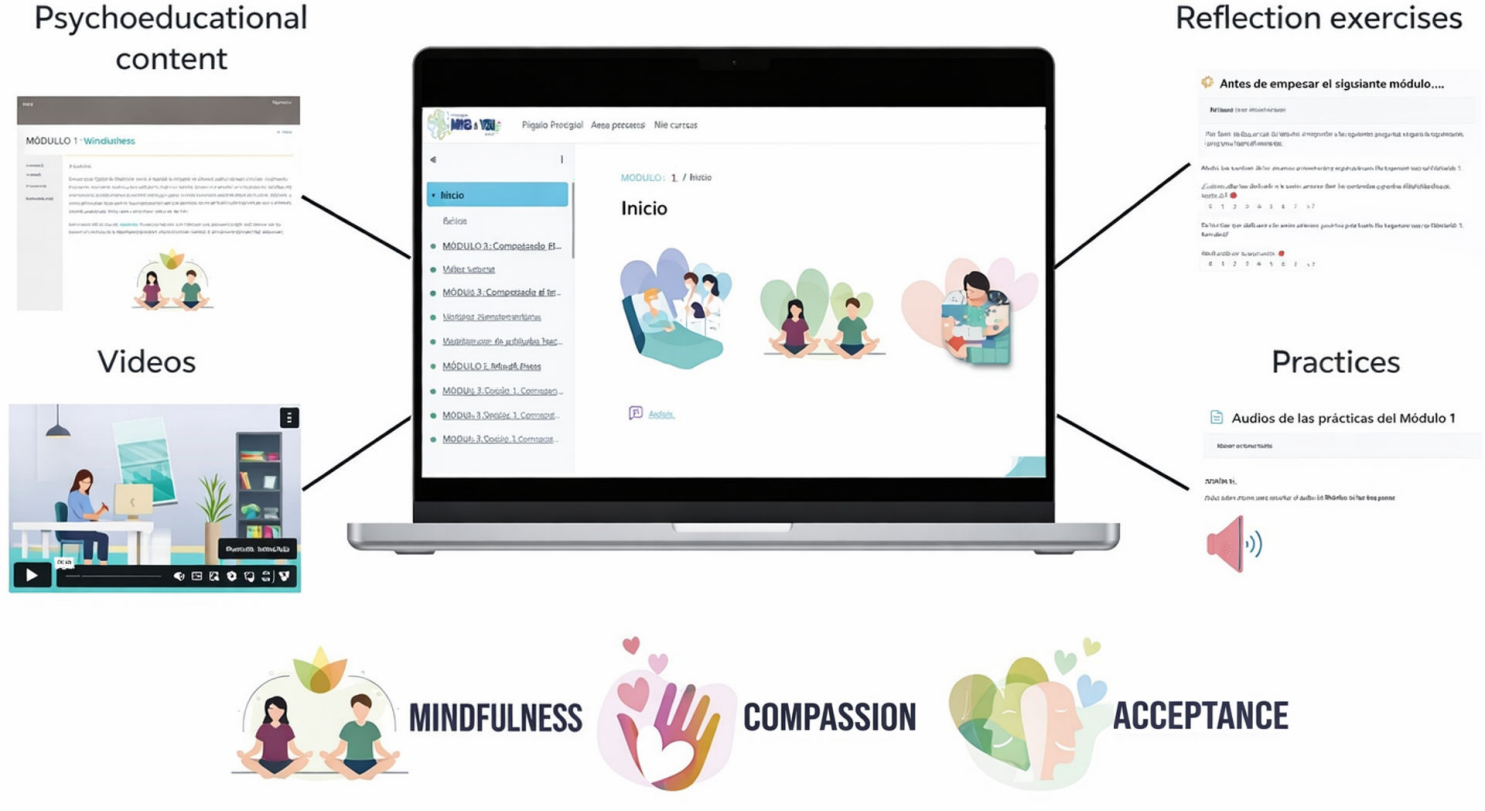
MINDxYOU program.

The MINDxYOU program is a self-guided web-based intervention delivered through a digital platform. It consists of 4 modules based on third wave psychotherapies (mindfulness, compassion, and acceptance), which have demonstrated effectiveness in reducing stress and promoting mental health [11]. Each module combines psychoeducational content, formal and informal practices, and experiential reflection exercises (Figure 1). The first module introduces the foundations of mindfulness and present-moment awareness. The second module focuses on cultivating a compassionate attitude toward oneself and others, addressing self-criticism and self-care. The third module explores the acceptance of difficult thoughts and emotions, promoting a more flexible relationship with internal experience. Finally, the fourth module integrates previous learning and focuses on applying it to work and personal contexts, with strategies to maintain long-term benefits. The intervention is designed to be completed over 8 weeks (2 weeks per module), with a flexible approach that allows users to adapt the pace to their needs.

Its content is adapted from validated programs such as Mindfulness-Based Stress Reduction [27], “Smiling is fun” [28], and Attachment-Based Compassion Therapy [29], incorporating specific examples from the health care context [11]. It includes formal practices (audio recordings) and informal ones (healthy habits such as exercise, nutrition, sleep, and socialization), promoting the integration of meditation into daily routines and continued practice after completing the program. Further information about the intervention can be found in the study protocol [23].

### Measures

As part of the MINDxYOU project, participants were asked to provide sociodemographic information (age and gender) and job-related details (profession, health care center, type of contract, and whether they were residents), as well as to complete the following questionnaires.

### Primary Outcome

The 10-item Perceived Stress Scale (PSS-10) [30] was the primary outcome of this study. It is a 10-item questionnaire that measures how unpredictable, uncontrollable, and overloaded the individual has felt their life to be over the previous month on a 5-point Likert-type scale. Scores range between 0 and 40, with higher scores reflecting higher levels of perceived stress. Our study made use of the Spanish adaptation [31], and the PSS for our sample showed very high internal consistency (αT1=0.89 and αT2-T5=0.91).

### Secondary Outcomes

The Patient Health Questionnaire (PHQ-9) [32] is a 9-item scale that rates the frequency of depressive symptoms during the previous 2 weeks using a Likert-type scale (from 0=not at all to 3=nearly every day). The total score ranges between 0 and 27, with higher scores indicating higher severity of depression. The Spanish adaptation of the PHQ-9 was used for this study [33], and we observed good internal consistency in our sample (αT1=0.83, αT2=0.88, αT3=0.86, αT4=0.89, and αT5=0.87).

The Generalized Anxiety Disorder-7 (GAD-7) [34] is a 7-item, self-report measure to assess the intensity of anxiety symptoms over the past 2 weeks. Using a 4-point Likert-type scale, the total score can range between 0 and 21, with higher values indicating more severe anxiety symptoms. The Spanish version of the GAD-7 [35] was used for this study, and excellent psychometric properties were observed in our sample (αT1=0.89, αT2=0.92, αT3=0.90, αT4=0.92, and αT5=0.91).

The Brief Symptom Inventory-18 (BSI-18) [36] is a 5-point Likert-type scale designed to offer rapid screening for the symptoms of psychological disorders (somatization, depression, and anxiety). Scores on the 18 items are summarized on the Global Severity Index, which ranges from 0 to 72, with higher scores reflecting more severe conditions. The Spanish version of the BSI-18 was used [37] and showed excellent internal consistency for our sample (αT1=0.90, αT2-T3=0.92, αT4=0.94, and αT5=0.92).

### Process Variables

Resilience was measured using the 10-item version of the Connor-Davidson Resilience Scale (CD-RISC) [38], a 10-item scale whose total score ranges between 0 and 40, with higher scores indicating higher levels of resilience. Mindfulness was assessed using the Five Facet Mindfulness Questionnaire-15 (FFMQ-15) [14] which assesses 5 dimensions: observing, describing, acting with awareness, nonjudging of inner experience, and not-reacting to inner experience. Each subscale comprises 3 items rated on a 5-point Likert scale, and mean scores are computed, with higher means denoting greater mindfulness in each domain.

Both self-compassion and compassion for others were assessed using the Sussex-Oxford Compassion Scales [39], which separately evaluate self-compassion and compassion for others using 20 items per scale. Scores range from 20 to 100, with higher totals indicating stronger levels of compassion. To assess psychological acceptance (vs experiential avoidance), the Acceptance and Action Questionnaire-II (AAQ-II) [40] was used. This 7-item instrument uses a 7-point Likert scale and provides a total score between 7 and 49, where higher scores correspond to greater experiential avoidance and thus lower acceptance.

The Spanish validated version of the CD-RISC [41], FFMQ-15 [42], Sussex-Oxford Compassion Scales [43], and AAQ-II [44] were applied in this study. These instruments demonstrated good to excellent internal consistency (*α*≥.80 across all time points), with the exception of several FFMQ subscales: Observing (αT1=0.74, αT2=.55, αT3=0.70, αT4–T5=0.74), Describing (αT1, T2, T5=0.77), and Nonreactivity (αT1=0.71, αT2–T3=0.58, αT4=.60, αT5=0.71).

### Statistical Analysis

Descriptive data analyses were conducted to characterize the sample, reporting frequencies and percentages for categorical variables and means and standard deviations for continuous variables. To estimate the indirect effect, a nonparametric bootstrap resampling procedure with 1000 iterations was performed, using parallel computing to optimize performance. In each bootstrap iteration, a random sample with replacement was drawn from the dataset, and 2 linear mixed-effects models with a random intercept for each participant were fitted to account for within-subject variability. The first model estimated the effect of the independent variable (intervention) on the mediator (Resilience, Mindfulness, Compassion, and Acceptance), while the second model evaluated the effect of the mediator on the primary outcome variable (perceived stress) and the secondary outcomes (depression, anxiety, and general psychological symptoms). The models were adjusted for the following covariates: cluster, gender, profession, age, type of contract, training status, and baseline level of the outcome variable. For the primary outcome, a multivariate analysis was also performed in which all mediators were included simultaneously. Additionally, sensitivity analyses were conducted using 2 multivariate models: 1 including only the mediators that were significant in the univariate analyses, and another including only 1 factor per mediator (Resilience, Mindfulness, Compassion, and Acceptance), selecting the most significant factor when multiple dimensions existed within the same mediator. The indirect effect was calculated as the product of the estimated coefficients from each bootstrap iteration. The direct effect of Treatment on the outcome variable was also estimated separately. A 95% CI for the indirect effect was derived from the 2.5th and 97.5th percentiles of the bootstrap distribution. Statistical significance was assessed via a 2-tailed test, with the *P* value calculated as the proportion of bootstrap samples in which the indirect effect crossed zero. To examine the temporal precedence of the most relevant mechanisms, additional time-lagged analyses were conducted. In these models, stress levels (PSS) at the subsequent time point were predicted from the prior value of the mediator, controlling for prior stress, intervention condition, time, and random effects for participant and cluster. These models were estimated specifically for the mediators that showed a more consistent association with stress in the concurrent analyses, namely, resilience and the FFMQ Observe facet. This approach makes it possible to assess whether higher levels of resilience or mindfulness at 1 measurement precede lower levels of stress at the next, providing complementary evidence regarding the potential temporal relationship between these psychological processes and stress reduction. All analyses were performed using R, with the *lme4* package for mixed-effects modeling and *future.apply* for parallel processing [45].

### Ethical Considerations

The research ethics committee of the Autonomous Community of Aragón and the Málaga Provincial Research Ethics Committee approved the study protocol in July 2022 (PI22/341). All study procedures complied with the Declaration of Helsinki and its most recent amendments. Participants provided written informed consent after being informed about the study, its potential risks, and their right to withdraw at any time. No financial compensation was offered for participation. Data confidentiality was ensured in accordance with Spain’s Organic Law 3/2018 on Data Protection and the EU General Data Protection Regulation (GDPR), using a dual-system infrastructure to keep personal data separate from clinical records. The trial was registered at ClinicalTrials.gov (NCT05436717, June 29, 2022) and reported following the CONSORT (Consolidated Standards of Reporting Trials) extension for stepped-wedge cluster randomized trials and the CONSORT-eHEALTH checklist.

## Results

### Sample Characteristics

**Figure 2.**
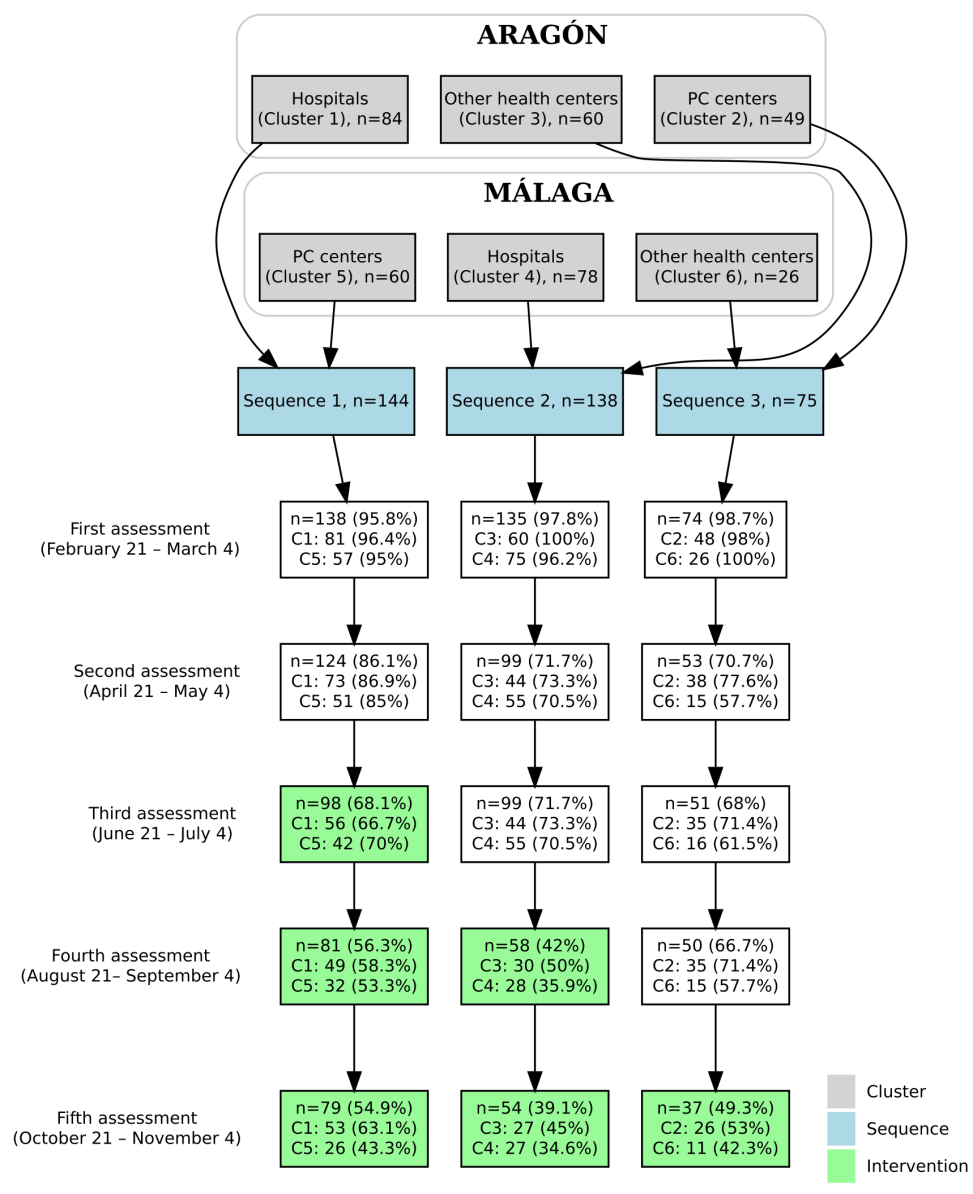
Randomization flowchart. PC: primary care center.

The final sample consisted of 347 HCPs distributed across 6 clusters (Figure 2). The majority were women (297/347, 85.6%), and the mean age was 45.01 years (SD 11.17). Most participants had a university-level education (304/347, 87.6%), although the proportion varied significantly between clusters (*P*<.001). A total of 260 participants (74.9%) were married or in a stable relationship. Regarding employment status, 283 professionals (81.6%) had a nontemporary contract, with significant variation across clusters (*P*=.01). In terms of professional roles, physicians (147/347, 42.4%) and nurses (93/347, 26.8%) were the most represented groups, followed by other HCPs (107/347, 30.8%); occupational distribution differed significantly across clusters (*P*<.001).

As for the baseline levels of perceived stress (PSS-10), the sample showed a mean score of 16.88 (SD 6.33), with no statistically significant differences across clusters (*P*=.07). Mean scores for the secondary outcomes were: PHQ-9=6.25 (SD 4.36), GAD-7=7.05 (SD 4.23), and BSI-18 Global Severity Index=12.34 (SD 9.88), none of which differed significantly by cluster (*P*>.05). Full descriptive data by cluster are presented in Table 1.

Of the 347 HCPs included in the analyses, the MINDxYOU program was initiated by 229 participants (66%), of whom 112 (48.9%) met the completer criterion, defined as having finished at least 3 of the 4 core modules. Adherence to the intervention was therefore moderate, consistent with previous findings in self-guided web-based programs.

**Table 1.** Baseline characteristics of the study sample. Missing values were found in the next variables: CD-RISC (1 in cluster 5), FFMQ (1 in cluster 4, 2 in cluster 5), SOCS-Others (1 in cluster 5), SOCS-Self (1 in cluster 4, 1 in cluster 5).

	Total sample (n=347)	Cluster 1 (n=81)	Cluster 2 (n=48)	Cluster 3 (n=60)	Cluster 4 (n=75)	Cluster 5 (n=57)	Cluster 6 (n=26)	Statistics	*P* value
Sociodemographic characteristics^a^									
Sex, n of females (%)	297 (85.6)	73 (90.1)	44 (91.7)	55 (91.7)	59 (78.7)	44 (75.9)	22 (84.6)	11.79 (5)^b^	.04
Age (years), mean (SD)	45.01 (11.17)	44.95 (10.42)	47.60 (12.24)	44.27 (10.23)	42.81 (10.03)	49.81 (11.32)	38.00 (11.62)	5.66 (5, 341)^c^	<.001
Work-related aspects								14.69 (5)^b^	.01
Type of contract, n (%)									
Temporary contract, n (%)	64 (18.4)	11 (13.6)	8 (16.7)	7 (11.7)	25 (33.3)	9 (15.8)	4 (15.4)		
Nontemporary contract	283 (81.6)	70 (13.6)	40 (83.3)	53 (88.7)	50 (66.7)	48 (84.2)	22 (84.6)		
Occupation, n (%)								72.72 (10)^b^	<.001
Physician	147 (42.4)	37 (45.7)	32 (66.7)	14 (23.3)	36 (48)	19 (33.3)	2 (7.7)		
Nurse	93 (26.8)	18 (22.2)	14 (29.2)	11 (18.3)	26 (34.7)	26 (45.6)	5 (19.2)		
Other	107 (30.8)	26 (32.1)	2 (4.2)	35 (58.3)	13 (17.3)	12 (21.1)	19 (73.1)		
Trainee, n (%)	34 (9.8)	9 (10.7)	9 (18.4)	—	11 (14.1)	5 (8.3)	—	15.64 (5)^b^	.008
Main outcome variable, mean (SD)									
PSS^d^ (score range 0‐40)	16.88 (6.33)	17.98 (6.35)	18.33 (6.02)	16.42 (6.13)	15.29 (5.64)	16.53 (7.09)	17.27 (6.75)	2.09 (5, 341)^c^	.07
Secondary outcome variables, mean (SD)									
PHQ-9^e^ (score range 0‐27)	6.25 (4.36)	6.81 (4.01)	6.65 (4.46)	6.00 (4.42)	5.40 (4.24)	6.35 (4.93)	6.58 (4.17)	0.99 (5, 341)^c^	.42
GAD-7^f^ (score range 0‐21)	7.05 (4.23)	7.72 (3.79)	7.46 (3.74)	6.88 (4.41)	6.11 (4.05)	6.89 (4.96)	7.62 (4.51)	1.37 (5, 341)^c^	.23
BSI-18^g^									
Somatization (score range 0‐24)	3.04 (3.50)	2.99 (3.43)	3.40 (3.44)	2.82 (3.17)	2.60 (3.30)	3.40 (3.34)	3.58 (5.20)	0.63 (5, 341)^c^	.68
Depression (score range 0‐24)	4.50 (4.18)	5.15 (4.30)	5.23 (4.53)	3.68 (3.63)	3.60 (3.57)	4.60 (4.49)	5.46 (4.74)	2.16 (5, 341)^c^	.06
Anxiety (score range 0‐24)	4.79 (3.84)	5.31 (3.84)	5.58 (3.66)	4.38 (3.41)	4.16 (3.81)	4.60 (4.03)	4.92 (4.57)	1.29 (5, 341)^c^	.27
GSI^h^ (score range 0‐72)	12.34 (9.88)	13.44 (9.74)	14.21 (9.74)	10.88 (8.80)	10.36 (9.25)	12.60 (10.30)	13.96 (12.86)	1.57 (5, 341)^c^	.17
Mediation variables, mean (SD)									
CD-RISC^i^ (score range 0‐40)	27.31 (6.79)	26.36 (7.01)	25.65 (6.58)	27.37 (7.13)	28.83 (6.35)	27.98 (6.55)	27.38 (6.98)	1.77 (5, 340)^c^	.12
FFMQ-15^j^ (score range 1-5)									
Observing	2.84 (0.86)	2.70 (0.74)	2.73 (0.87)	2.73 (0.85)	2.98 (0.89)	3.12 (0.87)	2.81 (0.98)	2.34 (5, 338)^c^	.04
Describing	3.54 (0.84)	3.55 (0.79)	3.33 (0.96)	3.52 (0.96)	3.65 (0.75)	3.55 (0.82)	3.59 (0.71)	0.88 (5, 338)^c^	.50
Acting with awareness	3.26 (0.91)	3.02 (0.86)	3.13 (0.97)	3.39 (0.92)	3.47 (0.84)	3.35 (0.84)	3.17 (1.05)	2.55 (5, 338)^c^	.03
Nonjudging	3.80 (0.87)	3.56 (0.88)	3.78 (0.82)	3.84 (0.87)	4.00 (0.90)	3.92 (0.83)	3.62 (0.83)	2.50 (5, 338)^c^	.03
Nonreacting	3.01 (0.85)	2.92 (0.78)	2.91 (0.67)	3.06 (0.85)	3.05 (0.92)	3.03 (0.95)	3.23 (0.91)	0.73 (5, 338)^c^	.60
SOCS^k^ (score range 20-100)									
Compassion for others	61.90 (8.77)	61.56 (9.06)	58.67 (9.41)	62.80 (2.67)	64.00 (7.44)	61.82 (8.24)	60.92 (8.94)	2.43 (5, 340)^c^	.04
Self-compassion	53.53 (10.24)	50.96 (9.27)	51.70 (10.79)	53.30 (10.90)	56.95 (9.70)	53.88 (10.01)	55.00 (10.42)	3.20 (5, 339)^c^	.008
AAQ-II^l^ (score range 7-49)	20.89 (8.33)	22.77 (7.61)	21.79 (5.59)	20.75 (8.35)	18.91 (6.69)	20.60 (8.62)	20.12 (7.52)	1.87 (5, 341)^c^	.10

a Effects that remained statistically significant (ie, *P*<.05) after applying the Benjamini-Hochberg correction for multiple tests.

b Chi-square (*df*).

c *F* test (*df*).

d PSS: Perceived Stress Scale.

e PHQ-9: Patient Health Questionnaire-9.

f GAD-7: Generalized Anxiety Disorder-7.

g BSI-18: Brief Symptom Inventory-18.

h GSI: Global Severity Index.

i CD-RISC: Connor-Davidson Resilience Scale.

j FFMQ-15: Five Facet Mindfulness Questionnaire-15.

k SOCS: Sussex-Oxford Compassion Scale.

l AAQ-II: Acceptance and Action Questionnaire-II.

### Mediation Analysis for Perceived Stress

**Figure 3.**
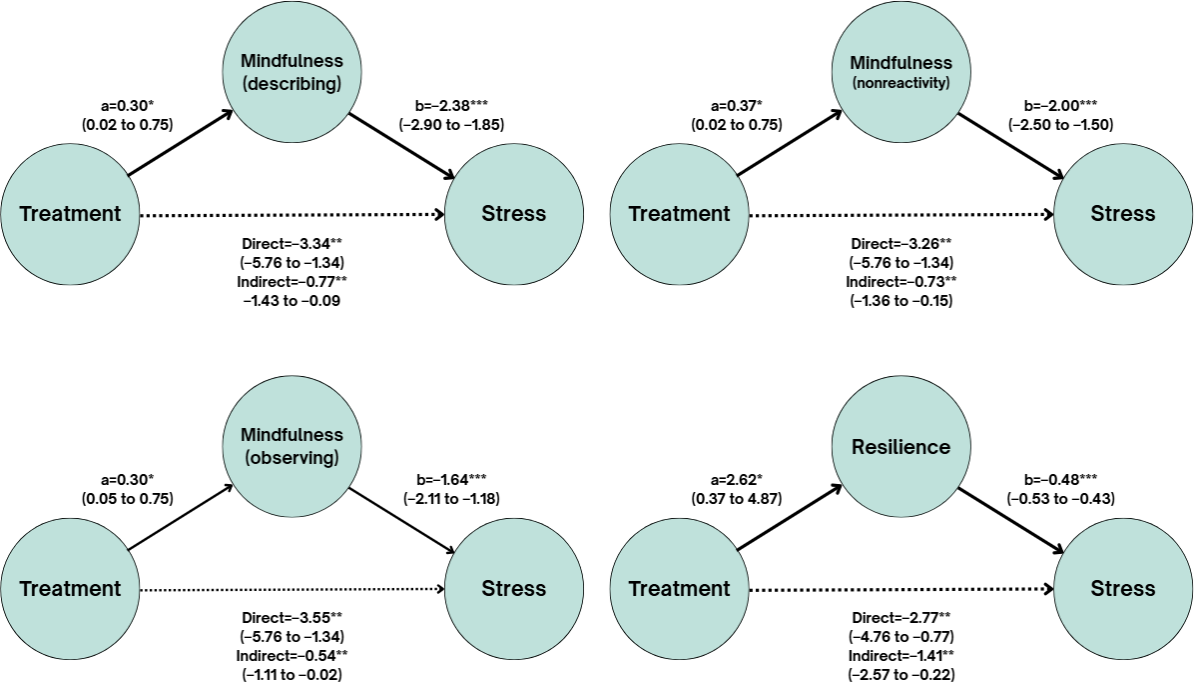
Path model of treatment effects on stress. *P<.05, **P<.01, and ***P<.001 path coefficients. 95% CIs are in parentheses.

In all mediation models, there was a significant direct effect of the intervention on levels of perceived stress. Significant indirect effects were observed through resilience (β=−1.41; *P*=.02), as well as through the mindfulness facets “Observing” (β=−0.54; *P*=.04), “Describing” (β=−0.77; *P*=.02), and “Nonreacting” (β=−0.73; *P*=.01). In all these cases, the intervention had a significant effect on the mediating variables, and the mediators had a significant effect on stress. No significant mediation effects were found for the mindfulness facets “Acting with awareness” and “Nonjudging,” nor for compassion (self-compassion or compassion for others) or acceptance. Further details on the mediation models are presented in Table 2. A graphical summary of the significant models is shown in Figure 3.

In the multivariate analysis, where all mediating variables were entered into the model simultaneously, only resilience showed a significant indirect effect on perceived stress (β=−0.803; *P*=.001). Detailed results can be found in Table 3. In the other multivariate models, resilience was also the only significant mediator, with comparable values. Further details of these models are presented in Tables S1 and S2 in Multimedia Appendix 1.

**Table 2.** Mediation effect of different variables between MINDxYOU intervention and perceived stress.

Mediation effect of different variables	β (95% CI)	SE	*P* value
Resilience			
a (treatment → resilience)	2.62 (0.37 to 4.87)	1.14	.02
b (resilience → stress)	−0.48 (−0.53 to −0.43)	0.03	<.001
Indirect effect (a×b)	−1.41 (−2.57 to −0.22)	0.59	.02
Direct effect (treatment → stress)	−2.77 (−4.76 to −0.77)	1.01	.007
Mindfulness (observing factor)			
a (treatment → observing)	0.30 (0.05 to 0.75)	0.15	.04
b (observing → stress)	−1.64 (−2.11 to −1.18)	0.23	<.001
Indirect effect (a×b)	−0.54 (−1.11 to −0.02)	0.29	.04
Direct effect (treatment → stress)	−0.55 (−5.76 to −1.34)	1.13	.002
Mindfulness (describing factor)			
a (treatment → describing)	0.30 (0.02 to 0.75)	0.12	.01
b (describing → stress)	−2.38 (−2.9 to −1.85)	0.25	<.001
Indirect effect (a×b)	−0.77 (−1.43 to −0.09)	0.34	.02
Direct effect (treatment → stress)	−3.34 (−5.76 to −1.34)	1.13	.003
Mindfulness (act factor)	−0.01 (−0.30 to 0.28)	0.15	. 95
a (treatment → act)	−0.01 (−0.3 to 0.28)	0.15	.95
b (act → stress)	−2.2 (−2.66 to −1.74)	0.24	0
Indirect effect (a×b)	0.03 (−0.67 to 0.72)	0.35	.96
Direct effect (treatment → stress)	−4.03 (−6.21 to −1.85)	1.11	0
Mindfulness (nonjudging factor)			
a (treatment → nonjudging)	0.01 (−0.28 to 0.3)	0.15	.92
b (nonjudging → stress)	−3 (−3.5 to −2.5)	0.22	<.001
Indirect effect (a×b)	−0.09 (−0.98 to 0.85)	0.47	.85
Direct effect (treatment → stress)	−4.02 (−5.76 to −1.34)	1.07	<.001
Mindfulness (nonreacting factor)			
a (treatment → nonreacting)	0.37 (0.02 to 0.75)	0.15	.01
b (nonreacting → stress)	−2 (−2.5 to −1.5)	0.24	<.001
Indirect effect (a×b)	−0.73 (−1.36 to −0.15)	0.31	.01
Direct effect (treatment → stress)	−3.26 (−5.76 to −1.34)	1.14	.004
Compassion for others			
a (treatment → compassion for others)	−0.01 (−2.5 to 2.5)	1.41	.99
b (compassion for others → stress)	−0.10 (−0.15 to −0.05)	0.02	<.001
Indirect effect (a×b)	0.03 (−0.26 to 0.31)	0.14	.82
Direct effect (treatment → stress)	−3.95 (−5.76 to −1.34)	1.16	<.001
Self-compassion			
a (treatment → self-compassion)	2.41 (−1.05 to 5.87)	1.77	.17
b (self-compassion → stress)	−0.22 (−0.25 to −0.18)	0.02	<.001
Indirect effect (a×b)	−0.55 (−1.37 to 0.40)	0.45	.23
Direct effect (treatment → stress)	−3.44 (−5.58 to −1.30)	1.09	.002
Acceptation			
a (treatment → acceptation)	−0.35 (−2.89 to 2.19)	1.30	.78
b (acceptation → stress)	.43 (0.38 to 0.48)	0.02	<.001
Indirect effect (a×b)	−0.22 (−1.39 to 0.90)	0.58	.70
Direct effect (treatment → stress)	−3.81 (−5.78 to −1.83)	1.01	<.001

**Table 3.** Multivariate mediation analysis.

Mediation	β (95% CI)	SE	*P* value
Resilience	−0.803 (−1.622 to –0.135)	0.369	.01
Mindfulness (bserving factor)	−0.135 (−0.365 to 0.013)	0.101	.11
Mindfulness (describing factor)	0.052 (−0.142 to 0.290)	0.106	.60
Mindfulness (nonreacting)	−0.110 (−0.360 to 0.059)	0.103	.23
Mindfulness (nonjudging factor)	−0.016 (−0.319 to 0.314)	0.152	.88
Mindfulness (act factor)	−0.005 (−0.197 to 0.177)	0.088	.98
Compassion (compassion for others)	−0.010 (−0.203 to 0.173)	0.094	.94
Compassion (self-compassion)	−0.102 (−0.343 to 0.066)	0.110	.27
Acceptation	−0.105 (−0.649 to 0.450)	0.273	.65

### Mediation Analysis for Depression

In all mediation models, there was a significant direct effect of the intervention on depression levels. Significant indirect effects were identified through resilience (β=−0.97; *P*=.01), as well as through the mindfulness facets “Observing” (β=−0.35; *P*=.04), “Describing” (β=−0.50; *P*=.03), and “Nonreacting” (β=−0.50; *P*=.01). In all cases, the intervention had a significant effect on the mediators, which in turn were associated with reductions in depressive symptoms. Nonsignificant mediation effects were observed for the facets “Acting with awareness” and “Nonjudging,” nor for compassion (self-compassion or compassion for others) or acceptance. Additional information on the depression models is provided in Table S3 and Figure S1 in Multimedia Appendix 1.

### Mediation Analysis for Anxiety

In all mediation models, there was a significant direct effect of the intervention on anxiety levels, as well as indirect effects through several mediating mechanisms. Significant indirect effects were identified through resilience (β=−0.88; *P*=.03), and through the mindfulness facets “Observing” (β=−0.34; *P*=.03), “Describing” (β=−0.47; *P*=.01), and “Nonreacting” (β=−2.13; *P*=.007). These mediating variables were significantly associated with lower levels of anxiety (β effects, *P*<.001). No mediation effects were found through the other variables. Further information on the anxiety models is presented in Table S4 and Figure S2 in Multimedia Appendix 1.

### Mediation Analysis for General Psychological Symptoms

Lastly, for general psychological symptoms, no significant direct effect of the intervention on the outcome variable was observed in most models, except for one. However, significant indirect effects were identified through resilience (β=−1.94; *P*=.01), and through the mindfulness facets “Observing” (β=−0.61; *P*=.04), “Describing” (β=−1.13; *P*=.01), and “Nonreacting” (β=−0.80; *P*=.02). Further information on the mediation models for psychological symptoms is presented in Table S5 and Figure S3 in Multimedia Appendix 1.

### Post Lagged Analysis

In the lagged analyses, the results showed that higher resilience at the previous assessment significantly predicted a subsequent reduction in stress (β=–0.21; *P*<.001). In contrast, the FFMQ Observing facet did not show a significant effect on later stress levels (β=–0.13; *P*=.66). The details of the lagged analysis are shown in Table S6 in Multimedia Appendix 1.

## Discussion

### Principal Findings

This study explored the psychological mechanisms through which the MINDxYOU digital program may reduce stress and psychological distress in HCPs. Specifically, it analyzed whether variables such as resilience, mindfulness facets, compassion, and acceptance mediated the impact of the intervention on stress, anxiety, depression, and general psychological symptoms.

This mediation-based approach builds upon previous evidence demonstrating the clinical effectiveness of MINDxYOU [24] and aligns with prior studies emphasizing the role of third wave psychotherapy components in improving mental health outcomes [11,12]. Recent systematic reviews have also highlighted the potential of brief digital interventions based on mindfulness and acceptance for health care personnel exposed to high levels of stress, burnout, or emotional exhaustion [46,47].

Regarding mechanisms of change, resilience emerged as the most consistent and robust mediator of the intervention’s effect, both in univariate and multivariate models. This finding aligns with theoretical models suggesting that third wave psychotherapies do not merely reduce symptomatology but also strengthen transdiagnostic psychological resources that support adaptive coping in high-demand environments [21,48]. The sustained significance of resilience in sensitivity analyses further supports its role as a primary mediating pathway. Several studies have documented improvements in resilience following brief digital interventions in HCPs. For example, the Mindfulness-Oriented Professional Resilience program was shown to enhance resilience and reduce psychological distress among frontline workers [49,50]. Likewise, a randomized trial reported that a 28-day digital resilience training led to significant improvements in resilience and stress reduction [51]. These results reinforce the idea that cultivating resilience may be a key process in improving mental health outcomes through digital formats, particularly in occupational settings marked by chronic stress.

Significant mediating effects were observed for several mindfulness facets, particularly “Observing,” “Describing,” and “Nonreacting,” suggesting that these specific components may facilitate more effective emotional regulation. These skills allow for greater awareness of internal experiences without automatic judgments or impulsive reactions, thereby enabling more adaptive emotional processing [17,18]. The fact that these dimensions show differentiated and not always consistent effects highlights the importance of deconstructing the mindfulness construct to accurately assess its active mechanisms. Recent research has emphasized that these specific dimensions of mindfulness are differentially associated with reductions in stress and psychological symptoms among HCPs, especially when targeted through focused interventions [46,52,53].

In contrast, neither acceptance nor compassion (self-compassion or compassion for others) showed statistically significant mediating effects. While this result aligns with some studies that question their consistency as universal mediators [54], it may be influenced by several factors. For instance, these skills may require more extensive practice or a more specific context to fully unfold their therapeutic potential. In fact, acceptance and compassion processes often show more robust effects in interventions with higher therapeutic dosage and guided practice [55,56], whereas the brief, self-guided nature of the MINDxYOU program may offer a more limited opportunity for these abilities to consolidate. Therefore, the absence of significant mediation effects should be interpreted cautiously, as it may reflect the format and intensity of the intervention rather than the irrelevance of these mechanisms. Alternatively, they may play a more modulatory rather than mediating role, facilitating sustained engagement with the program rather than exerting direct symptomatic effects particularly in self-guided digital environments [57].

From an applied perspective, these findings have important implications. On one hand, they suggest the value of incorporating explicit modules focused on strengthening resilience in interventions targeting HCPs, especially in post-pandemic contexts characterized by high emotional overload and increased psychosocial risk [1]. On the other hand, they indicate that not all facets of mindfulness hold the same clinical relevance, prompting a reconsideration of generic approaches and supporting a more selective, evidence-based implementation of the most effective components.

Finally, these results contribute to the growing body of literature on mechanisms of change in digital psychological interventions, highlighting that the online format can be effective not only in reducing symptoms but also in promoting positive psychological processes. The identification of specific mediators such as resilience and certain mindfulness facets provides valuable evidence for optimizing the design, adaptation, and personalization of future digital interventions aimed at improving the well-being of HCPs [47,58].

### Limitations

This study has several limitations. First, the mediating variables were assessed at specific time points, which limits the ability to analyze the dynamic evolution of change processes. Additionally, only certain theoretical mechanisms were considered (resilience, mindfulness, compassion, and acceptance), excluding others that may also be relevant, such as psychological flexibility or emotional regulation. Likewise, although the program included a module promoting healthy lifestyle habits (eg, sleep, physical activity, nutrition, and social interaction), behavioral changes in these areas were not assessed. As such, we cannot rule out the possibility that improvements in stress and emotional well-being were partly mediated by changes in daily health behaviors. Future studies should incorporate validated instruments to examine the potential mediating role of lifestyle-related factors. It is also important to note the exclusive use of self-report measures, which may be influenced by perception biases or social desirability. Furthermore, the study lacked a parallel control group, which restricts the ability to draw firm causal conclusions beyond the stepped-wedge design. The absence of a qualitative evaluation also limits the exploration of participants’ subjective experiences, acceptability of the intervention, and contextual implementation barriers. In addition, adherence to the MINDxYOU program was moderate, with approximately half of the participants meeting the completer criterion. Although this level of engagement is comparable to other self-guided digital interventions, the absence of detailed adherence data such as module-by-module completion rates, time spent on the platform, or reasons for drop-out limits a deeper understanding of participant engagement and its potential influence on treatment outcomes. Moreover, the sample may present a selection bias, as it consisted of HCPs who voluntarily chose to participate, possibly more motivated or familiar with such interventions, which may limit the generalizability of the findings. In addition, the study was conducted in only 2 regions of Spain (Aragón and Northeast Málaga), which may limit the external validity and generalizability of the findings to other health care systems or cultural contexts. Finally, it is worth noting that some mindfulness subscales showed modest internal consistency, which may have reduced the statistical sensitivity to detect associations with outcomes, suggesting measurement constraints could have partly contributed to null findings in these domains.

### Conclusions

Resilience emerged as the most consistent and robust mediating mechanism, suggesting that strengthening this capacity is a key pathway through which interventions based on third wave psychotherapy principles exert their therapeutic effect. Certain specific mindfulness facets, namely observing, describing, and nonreacting, also showed significant, though less consistent, mediating effects. In contrast, compassion and acceptance did not show a significant mediating role in this context. These findings highlight the importance of designing digital interventions that enhance core psychological resources such as resilience and suggest that focusing on specific mindfulness skills may further boost the benefits of such programs. This evidence provides valuable guidance for the development and optimization of future digital interventions aimed at promoting the well-being of HCPs.

## Supplementary material

10.2196/82905 Multimedia Appendix 1 Supplementary tables and figures.

10.2196/82905 Checklist 1 CONSORT checklist.

## Abbreviations

AAQ-II: Acceptance and Action Questionnaire-II
ACT: acceptance and commitment therapy
BSI-18: Brief Symptom Inventory-18
CD-RISC: Connor-Davidson Resilience Scale
CONSORT: Consolidated Standards of Reporting Trials
FFMQ-15: Five Facet Mindfulness Questionnaire-15
GAD-7: Generalized Anxiety Disorder-7
HCP: health care professional
PHQ-9: Patient Health Questionnaire-9
