# Supplementary material for: Resilience as a Mediator in a Web-Based Intervention (MINDxYOU) to Reduce Stress Among Health Care Professionals: Stepped-Wedge Cluster Randomized Trial
Source: JMIR Ment Health. 2026 Feb 9;13:e82905. doi: 10.2196/82905 (PMC12885452; doi:10.2196/82905)
Supplement: Multimedia Appendix 1 [file mental-v13-e82905-s001.docx]

**Table S1.** Indirect effects in the multivariate mediation analysis in which the significant variables of the univariate models have been included.

| **Mediation** | B | SE | CI Lower | CI Upper | p value |
| --- | --- | --- | --- | --- | --- |
| Resilience | -1.225 | 0.540 | -2.359 | -0.204 | 0.014 |
| Mindfulness (Observing Factor) | -0.128 | 0.103 | -0.371 | 0.022 | 0.124 |
| Mindfulness (Describing Factor) | -0.135 | 0.105 | -0.374 | 0.034 | 0.132 |
| Mindfulness (Non-Reacting Factor) | -0.196 | 0.129 | -0.509 | 0.004 | 0.058 |

**Table S2.** Indirect effects in the multivariate mediation analysis in which only one factor per mediator has been included.

| **Mediation** | B | SE | CI Lower | CI Upper | p value |
| --- | --- | --- | --- | --- | --- |
| Resilience | -0.888 | 0.402 | -1.787 | -0.146 | 0.014 |
| Mindfulness (Non-Reacting Factor) | -0.125 | 0.106 | -0.384 | 0.038 | 0.166 |
| Compassion (Self-Compassion) | -0.073 | 0.086 | -0.274 | 0.057 | 0.342 |
| Acceptation | -0.139 | 0.354 | -0.829 | 0.575 | 0.652 |

**Table S3.** Mediation effect of different variables between MindxYou intervention and Depression

| **Mediation Effect of Resilience** | B | SE | CI Lower | CI Upper | p value |
| --- | --- | --- | --- | --- | --- |
| a (Treatment→ Resilience) | 2.62 | 1.14 | 0.36 | 4.88 | 0.022 |
| b (Resilience → Depression) | -0.34 | 0.02 | -0.38 | -0.29 | 0.000 |
| Indirect Effect (a × b) | -0.97 | 0.42 | -1.81 | -0.20 | 0.018 |
| Direct Effect (Treatment → Depression) | -1.94 | 0.69 | -3.28 | -0.60 | 0.005 |
| **Mediation Effect of Mindfulness (Observing Factor)** | B | SE | CI Lower | CI Upper | p value |
| a (Treatment→ Observing) | 0.30 | 0.15 | 0.01 | 0.59 | 0.047 |
| b (Observing → Depression) | -1.09 | 0.17 | -1.42 | -0.76 | 0.000 |
| Indirect Effect (a × b) | -0.35 | 0.18 | -0.72 | -0.02 | 0.040 |
| Direct Effect (Treatment → Depression) | -2.47 | 0.78 | -4.00 | -1.00 | 0.002 |
| **Mediation Effect of Mindfulness (Describing Factor)** | B | SE | CI Lower | CI Upper | p value |
| a (Treatment→ Describing) | 0.30 | 0.12 | 0.06 | 0.54 | 0.016 |
| b (Describing → Depression) | -1.53 | 0.18 | -1.88 | -1.18 | 0.000 |
| Indirect Effect (a × b) | -0.50 | 0.23 | -0.98 | -0.06 | 0.030 |
| Direct Effect (Treatment → Depression) | -2.35 | 0.78 | -3.90 | -1.20 | 0.003 |
| **Mediation Effect of Mindfulness (Act) Factor)** | B | SE | CI Lower | CI Upper | p value |
| a (Treatment→ Act) | -0.01 | 0.15 | -0.30 | 0.28 | 0.950 |
| b (Act→ Depression) | -1.54 | 0.17 | -1.88 | -1.21 | 0.000 |
| Indirect Effect (a × b) | 0.01 | 0.22 | -0.41 | 0.45 | 0.942 |
| Direct Effect (Treatment → Depression) | -2.79 | 0.77 | -4.29 | -1.28 | 0.000 |
| **Mediation Effect of Mindfulness (Non Judge Factor)** | B | SE | CI Lower | CI Upper | p value |
| a (Treatment→ Non Judge) | 0.01 | 0.15 | -0.29 | 0.32 | 0.930 |
| b (Non Judge → Depression) | -1.98 | 0.16 | -2.32 | -1.63 | 0.000 |
| Indirect Effect (a × b) | -0.07 | 0.30 | -0.68 | 0.53 | 0.790 |
| Direct Effect (Treatment → Depression) | -0.07 | 0.30 | -0.68 | 0.53 | 0.790 |
| **Mediation Effect of Mindfulness (Non-Reacting Factor)** | B | SE | CI Lower | CI Upper | p value |
| a (Treatment→ Non-Reacting) | 0,37 | 0,15 | 0,02 | 0,72 | 0.020 |
| b (Non-Reacting → Depression) | -1,39 | 0,17 | -1,94 | -0,11 | 0.000 |
| Indirect Effect (a × b) | -0,5 | 0,21 | -0,94 | -0,11 | 0.010 |
| Direct Effect (Treatment → Depression) | -2,28 | 0,78 | -3,84 | -0,72 | 0.000 |
| **Mediation Effect of Compassion for others** | B | SE | CI Lower | CI Upper | p value |
| a (Treatment→ Compassion for others) | 0.01 | 1.41 | -2.78 | 2.80 | 0.996 |
| b (Compassion for others → Depression) | -0.07 | 0.02 | -0.10 | 0.01 | 0.126 |
| Indirect Effect (a × b) | -0.00 | 0.10 | -0.19 | 0.21 | 0.900 |
| Direct Effect (Treatment → Depression) | -2.77 | 0.80 | -4.32 | -1.23 | 0.001 |
| **Mediation Effect of Self-Compassion** | B | SE | CI Lower | CI Upper | p value |
| a (Treatment→ Self-Compassion) | 2.41 | 1.77 | -1.10 | 5.92 | 0.174 |
| b (Self-Compassion → Depression) | -0.15 | 0.01 | -0.18 | -0.12 | 0.000 |
| Indirect Effect (a × b) | -0.39 | 0.30 | -1.00 | 0.21 | 0.212 |
| Direct Effect (Treatment → Depression) | -2.40 | 0.75 | -3.90 | -0.90 | 0.001 |
| **Mediation Effect of Acceptation** | B | SE | CI Lower | CI Upper | p value |
| a (Treatment→ Acceptation) | -0.35 | 1.30 | -2.91 | 2.21 | 0.786 |
| b (Acceptation → Depression) | 0.32 | 0.02 | 0.28 | 0.36 | 0.000 |
| Indirect Effect (a × b) | -0.18 | 0.41 | -0.97 | 0.63 | 0.640 |
| Direct Effect (Treatment → Depression) | -2.64 | 0.69 | -4.01 | -1.27 | 0.000 |

**Table S4.** Mediation effect of different variables between MindxYou intervention and Anxiety

| **Mediation Effect of Resilience** | B | SE | CI Lower | CI Upper | p value |
| --- | --- | --- | --- | --- | --- |
| a (Treatment→ Resilience) | 2.62 | 1.14 | 0.38 | 4.86 | 0.022 |
| b (Resilience → Anxiety) | -0.32 | 0.02 | -0.35 | -0.28 | <0.001 |
| Indirect Effect (a × b) | -0.88 | 0.39 | -1.63 | -0.16 | 0.034 |
| Direct Effect (Treatment → Anxiety) | -1.82 | 0.70 | -3.19 | -0.44 | 0.009 |
| **Mediation Effect of Mindfulness (Observing Factor)** | B | SE | CI Lower | CI Upper | p value |
| a (Treatment→ Observing) | 0.30 | 0.15 | 0.00 | 0.60 | 0.047 |
| b (Observing → Anxiety) | -1.04 | 0.16 | -1.36 | -0.71 | 0.000 |
| Indirect Effect (a × b) | -0.34 | 0.18 | -0.71 | -0.02 | 0.038 |
| Direct Effect (Treatment → Anxiety) | -2.33 | 0.78 | -3.85 | -0.80 | 0.003 |
| **Mediation Effect of Mindfulness (Describing Factor)** | B | SE | CI Lower | CI Upper | p value |
| a (Treatment→ Describing) | 0.30 | 0.12 | 0.05 | 0.55 | 0.016 |
| b (Describing → Anxiety) | -1.42 | 0.18 | -1.77 | -1.07 | 0.000 |
| Indirect Effect (a × b) | -0.47 | 0.21 | -0.88 | -0.06 | 0.018 |
| Direct Effect (Treatment → Anxiety) | -2.23 | 0.77 | -3.75 | -0.72 | 0.004 |
| **Mediation Effect of Mindfulness (Act) Factor)** | B | SE | CI Lower | CI Upper | p value |
| a (Treatment→ Act) | -0.01 | 0.15 | -0.30 | 0.28 | 0.950 |
| b (Act→ Anxiety) | -1.55 | 0.17 | -1.87 | -1.22 | 0.000 |
| Indirect Effect (a × b) | 0.01 | 0.23 | -0.41 | 0.48 | 0.942 |
| Direct Effect (Treatment → Anxiety) | -2.64 | 0.76 | -4.13 | -1.14 | 0.001 |
| **Mediation Effect of Mindfulness (Non Judge Factor)** | B | SE | CI Lower | CI Upper | p value |
| a (Treatment→ Non Judge) | 0.01 | 0.15 | -0.29 | 0.31 | 0.927 |
| b (Non Judge → Anxiety) | -2.25 | 0.15 | -2.54 | -1.95 | 0.000 |
| Indirect Effect (a × b) | -0.06 | 0.34 | -0.70 | 0.65 | 0.822 |
| Direct Effect (Treatment → Anxiety) | -2.58 | 0.73 | -4.02 | -1.15 | 0.000 |
| **Mediation Effect of Mindfulness (Non-Reacting Factor)** | 0.37 | 0.15 | 0.07 | 0.67 | 0.016 |
| a (Treatment→ Non-Reacting) | -1.31 | 0.17 | -1.64 | -0.98 | 0.000 |
| b (Non-Reacting → Anxiety) | -0.47 | 0.19 | -0.84 | -0.10 | 0.018 |
| Indirect Effect (a × b) | -2.13 | 0.78 | -3.66 | -0.60 | 0.007 |
| Direct Effect (Treatment → Anxiety) | 0.37 | 0.15 | 0.07 | 0.67 | 0.016 |
| **Mediation Effect of Compassion for others** | B | SE | CI Lower | CI Upper | p value |
| a (Treatment→ Compassion for others) | -0.01 | 1.41 | -0.17 | 0.22 | 0.996 |
| b (Compassion for others → Anxiety ) | -0.07 | 0.02 | -0.10 | -0.03 | 0.000 |
| Indirect Effect (a × b) | 0.02 | 0.10 | -0.17 | 0.22 | 0.840 |
| Direct Effect (Treatment → Anxiety) | -2.60 | 0.79 | -4.15 | -1.05 | 0.001 |
| **Mediation Effect of Self-Compassion** | B | SE | CI Lower | CI Upper | p value |
| a (Treatment→ Self-Compassion) | 2.41 | 1.77 | -1.06 | 5.87 | 0.174 |
| b (Self-Compassion→ Anxiety) | -0.15 | 0.01 | -0.18 | -0.13 | 0.000 |
| Indirect Effect (a × b) | -0.39 | 0.30 | -0.97 | 0.18 | 0.190 |
| Direct Effect (Treatment → Anxiety) | -2.26 | 0.74 | -3.71 | -0.81 | 0.002 |
| **Mediation Effect of Acceptation** | B | SE | CI Lower | CI Upper | p value |
| a (Treatment→ Acceptation) | -0.35 | 1.30 | -1.02 | 0.74 | 0.786 |
| b (Acceptation → Anxiety) | 0.32 | 0.02 | 0.29 | 0.35 | 0.000 |
| Indirect Effect (a × b) | -0.16 | 0.44 | -1.02 | 0.74 | 0.674 |
| Direct Effect (Treatment →Anxiety) | -2.47 | 0.69 | -3.82 | -1.11 | 0.000 |

**Table S5.** Mediation effect of different variables between MindxYou intervention and Psychological Symptoms

| **Mediation Effect of Resilience** | B | SE | CI Lower | CI Upper | p value |
| --- | --- | --- | --- | --- | --- |
| a (Treatment→ Resilience) | 2.75 | 1.15 | 0.02 | 5.48 | 0.017 |
| b (Resilience → Psychological Symptoms) | -0.65 | 0.04 | -0.73 | -0.57 | 0.000 |
| Indirect Effect (a × b) | -1.94 | 0.81 | -3.49 | -0.26 | 0.018 |
| Direct Effect (Treatment → Psychological Symptoms) | -1.22 | 1.42 | -3.99 | 1.55 | 0.393 |
| **Mediation Effect of Mindfulness (Observing Factor)** | B | SE | CI Lower | CI Upper | p value |
| a (Treatment→ Observing) | 0.30 | 0.15 | 0.01 | 0.59 | 0.046 |
| b (Observing → Psychological Symptoms) | -1.86 | 0.33 | -2.51 | -1.21 | 0.000 |
| Indirect Effect (a × b) | -0.61 | 0.33 | -1.34 | -0.03 | 0.042 |
| Direct Effect (Treatment → Psychological Symptoms) | -2.53 | 1.64 | -5.73 | 0.67 | 0.122 |
| **Mediation Effect of Mindfulness (Describing Factor)** | B | SE | CI Lower | CI Upper | p value |
| a (Treatment→ Describing) | 0.30 | 0.12 | 0.01 | 0.59 | 0.015 |
| b (Describing → Psychological Symptoms) | -3.47 | 0.34 | -4.13 | -2.81 | 0.000 |
| Indirect Effect (a × b) | -1.13 | 0.50 | -2.14 | -0.15 | 0.016 |
| Direct Effect (Treatment → Psychological Symptoms) | -2.08 | 1.60 | -5.17 | 0.99 | 0.193 |
| **Mediation Effect of Mindfulness (Act Factor)** | B | SE | CI Lower | CI Upper | p value |
| a (Treatment→ Act) | -0.00 | 0.15 | -0.30 | 0.30 | 0.999 |
| b (Act→ Psychological Symptoms) | -2.72 | 0.33 | -3.35 | -2.09 | 0.000 |
| Indirect Effect (a × b) | 0.04 | 0.43 | -0.77 | 0.96 | 0.982 |
| Direct Effect (Treatment → Psychological Symptoms) | -3.02 | 1.60 | -6.17 | 0.14 | 0.060 |
| **Mediation Effect of Mindfulness (Non Judge Factor)** | B | SE | CI Lower | CI Upper | p value |
| a (Treatment→ Non Judge) | 0.03 | 0.15 | -0.26 | 0.32 | 0.860 |
| b (Non Judge → Psychological Symptoms) | -4.56 | 0.31 | -5.16 | -3.96 | 0.000 |
| Indirect Effect (a × b) | -0.17 | 0.72 | -1.56 | 1.26 | 0.800 |
| Direct Effect (Treatment → Psychological Symptoms) | -2.98 | 1.49 | -5.90 | -0.06 | 0.046 |
| **Mediation Effect of Mindfulness (Non-Reacting Factor)** | B | SE | CI Lower | CI Upper | p value |
| a (Treatment→ Non-Reacting) | 0.37 | 0.15 | 0.07 | 0.67 | 0.015 |
| b (Non-Reacting → Psychological Symptoms) | -2.23 | 0.34 | -2.90 | -1.56 | 0.000 |
| Indirect Effect (a × b) | -0.80 | 0.35 | -1.51 | -0.11 | 0.024 |
| Direct Effect (Treatment → Psychological Symptoms) | -2.23 | 1.64 | -5.50 | 1.04 | 0.175 |
| **Mediation Effect of Compassion for others** | B | SE | CI Lower | CI Upper | p value |
| a (Treatment→ Compassion for others) | 0.00 | 1.41 | -2.50 | 2.50 | 0.997 |
| b (Compassion for others → Psychological Symptoms ) | -0.15 | 0.03 | -0.41 | -0.08 | 0.000 |
| Indirect Effect (a × b) | 0.02 | 0.23 | -0.42 | 0.47 | 0.942 |
| Direct Effect (Treatment → Psychological Symptoms) | -2.98 | 1.65 | -6.00 | 1.50 | 0.071 |
| **Mediation Effect of Self-Compassion** | B | SE | CI Lower | CI Upper | p value |
| a (Treatment→ Self-Compassion) | 2.51 | 1.77 | -0.59 | 5.61 | 0.157 |
| b (Self-Compassion → Psychological Symptoms) | -0.31 | 0.02 | -0.36 | -0.27 | 0.001 |
| Indirect Effect (a × b) | -0.84 | 0.65 | -2.15 | 0.35 | 0.180 |
| Direct Effect (Treatment → Psychological Symptoms) | -2.23 | 1.54 | -5.26 | 0.80 | 0.149 |
| **Mediation Effect of Acceptation** | B | SE | CI Lower | CI Upper | p value |
| a (Treatment→ Acceptation) | -0.58 | 1.30 | -3.51 | 2.35 | 0.653 |
| b (Acceptation → Psychological Symptoms) | 0.64 | 0.03 | 0.60 | 0.67 | 0.001 |
| Indirect Effect (a × b) | -0.44 | 0.86 | -2.11 | 1.27 | 0.584 |
| Direct Effect (Treatment → Psychological Symptoms) | -2.69 | 1.39 | -5.70 | 0.33 | 0.053 |

**Table S6**. Lagged analysis of Resilience and Mindfulness on perceived stress

| **Lagged effect of Resilience** | B | SE | df | t | p |
| --- | --- | --- | --- | --- | --- |
| Resilience (CDRISC t-1) | -0.21 | 0.04 | 530 | - 4.79 | < 0.001 |
| Stress (PSS t-1) | 0.40 | 0.05 | 530 | 8.46 | < 0.001 |
| Intervention | -1.35 | 0.60 | 530 | -2.26 | 0024 |
| Time | 0.27 | 0.36 | 530 | 0.75 | 0.455 |
| **Lagged effect of Mindfulness (Observing)** | B | SE | df | t | p |
| Mindfulness (FFMQ t-1) | -0.13 | 0.30 | 530 | -0.44 | 0.660 |
| Stress (PSS t-1) | 0.54 | 0.04 | 530 | 14.36 | < 0.001 |
| Intervention | -1.45 | 0.61 | 530 | -2.38 | 0.018 |
| Time | 0.43 | 0.37 | 530 | 1.16 | 0.246 |

**
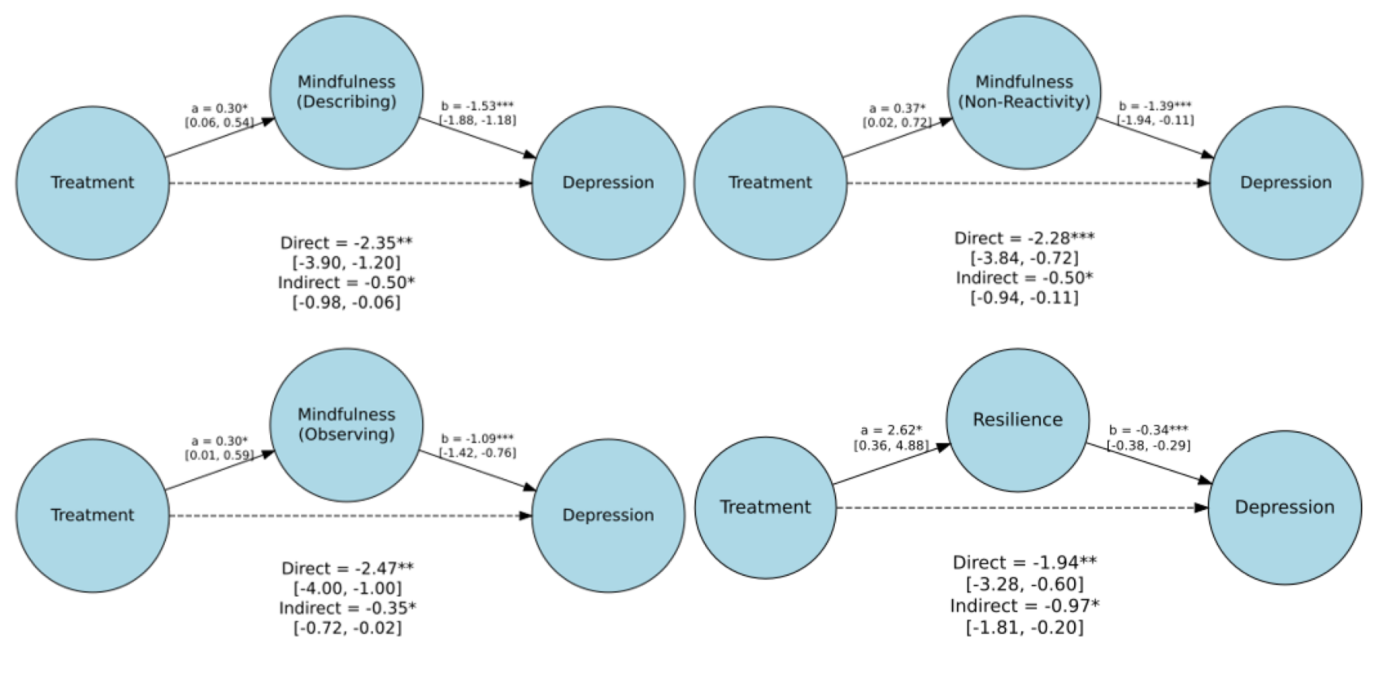
**

**Figure S1.** Path Model of Treatment Effects on Depression

Note. * means p value < .001. Path Coefficients (B). 95% Confidence Intervals (CI) are detailed between brackets.

**
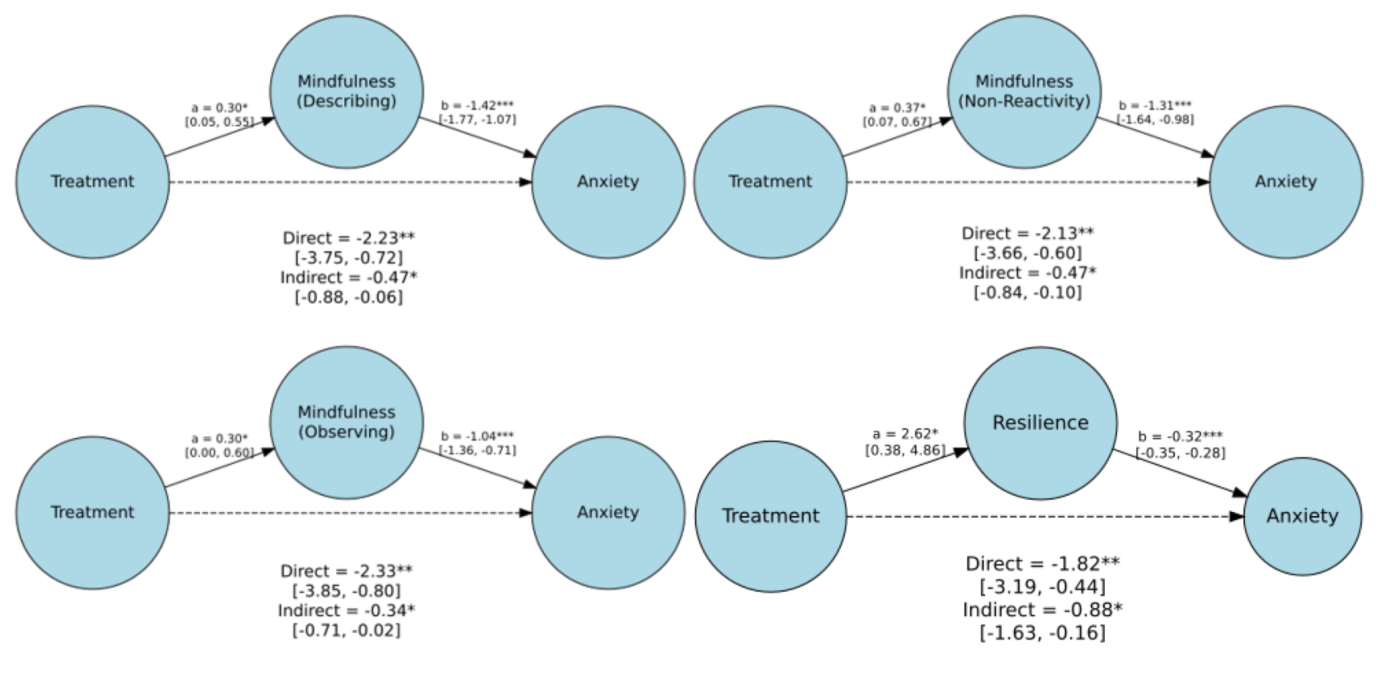
**

**Figure S2.** Path Model of Treatment Effects on Anxiety

Note. * means p value < .001. Path Coefficients (B). 95% Confidence Intervals (CI) are detailed between brackets.


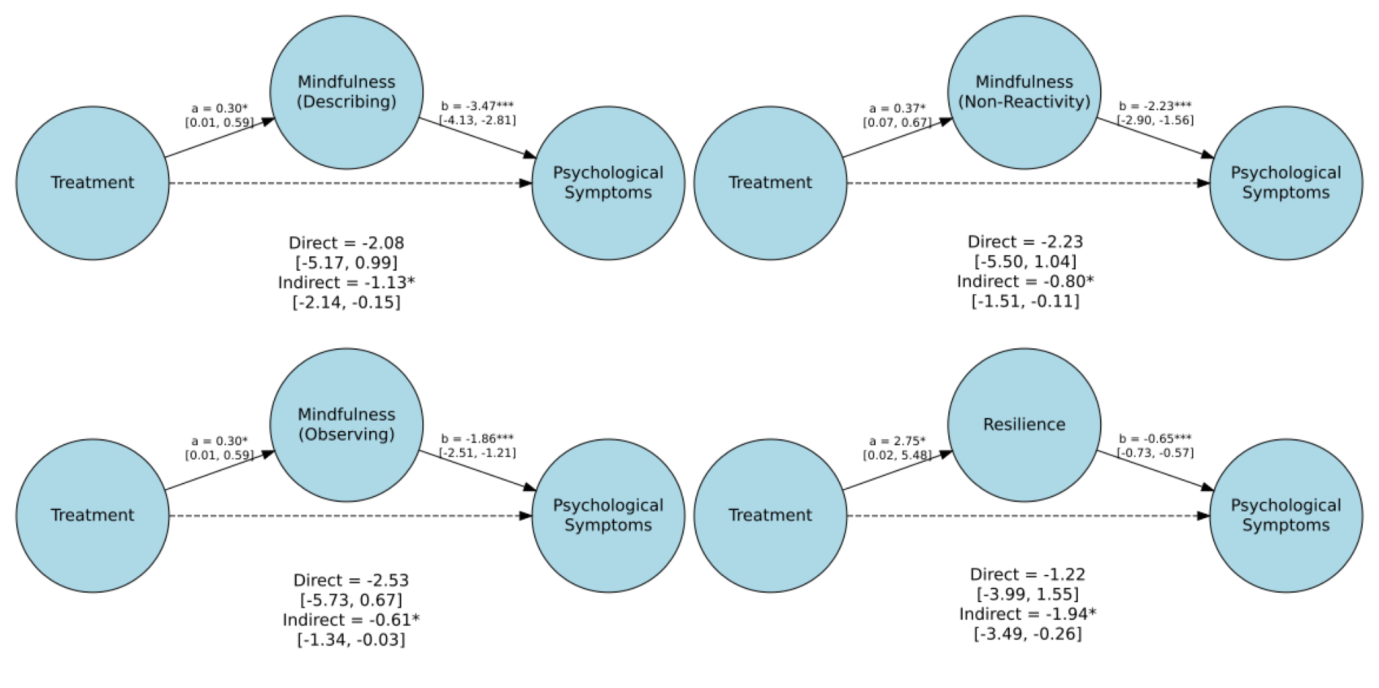


**Figure S3.** Path Model of Treatment Effects on Psychological Symptoms

Note. * means p value < .001. Path Coefficients (B). 95% Confidence Intervals (CI) are detailed between brackets.
